# Vaccine-Associated Paralytic Poliomyelitis and BCG-osis in an Immigrant Child with Severe Combined Immunodeficiency Syndrome — Texas, 2013

**Published:** 2014-08-22

**Authors:** Robert Trimble, Jane Atkins, Troy C. Quigg, Cara C. Burns, Gregory S. Wallace, Mary Thomas, Anil T. Mangla, Anthony J. Infante

**Affiliations:** 1 Department of Pediatrics, University of Texas Health Science Center, San Antonio, Texas; 2 Pediatrics Infectious Diseases, San Antonio, Texas; 3 Pediatric Blood and Marrow Transplantation, Methodist Physicians, San Antonio, Texas; 4 Division of Viral Diseases, CDC; 5 San Antonio Metropolitan Health District, San Antonio, Texas

Poliovirus transmission has been eliminated in most of the world through the use of inactivated poliovirus vaccine (IPV) and live, attenuated oral poliovirus vaccine (OPV). In the United States, use of OPV was discontinued by the year 2000 because of the potential for vaccine-associated paralytic polio (VAPP); an average of eight cases were reported each year in the United States during 1980–2000 (1). Polio eradication efforts in other parts of the world continue to rely on OPV to take advantage of transmission of poliovirus vaccine strains to unvaccinated persons in the population, lower cost, and ease of administration. In 2013, an infant aged 7 months who recently immigrated to the United States from India was referred to a hospital in San Antonio, Texas. The infant had fever, an enlarging skin lesion in the deltoid region with axillary lymphadenopathy, decreased activity, and inability to bear weight on the left leg, progressing to paralysis of the left leg over a 6-week period. Recognition of lymphopenia on complete blood count led to immune evaluation, which revealed the presence of severe combined immunodeficiency syndrome (SCIDS), an inherited disorder. A history of OPV and bacille Calmette-Guérin (BCG) vaccination in India led to the diagnoses of VAPP and BCG-osis, which were confirmed microbiologically. This report demonstrates the importance of obtaining a comprehensive clinical history in a child who has recently immigrated to the United States, with recognition that differing vaccine practices in other countries might require additional consideration of potential etiologies.

The last outbreak of polio caused by importation of wild poliovirus in the United States occurred in 1979 in an unvaccinated community (2). The last endemically acquired case of VAPP in the United States occurred in the same community in 1999 (2). In 2005, an unvaccinated U.S. resident was infected with polio vaccine virus in Costa Rica and subsequently developed VAPP (2). A case of immunodeficiency-associated vaccine-derived poliovirus (iVDPV) infection, without paralysis, was diagnosed in an unvaccinated child with SCIDS in 2005, but the source of the virus could not be definitively identified (3). A woman in Minnesota aged 44 years with long-standing common variable immunodeficiency died after developing VAPP in 2009 (4). She was probably infected when her child received OPV approximately 12 years earlier. Case reports and cohort studies from several countries other than the United States demonstrate the continued occurrence of iVDPVs and the need for ongoing surveillance (5).

BCG, a live vaccine strain of *Mycobacterium bovis*, is commonly used to prevent the spread and disease burden of tuberculosis (TB) but is not used in the United States because of the low prevalence of TB in the general population and the fact that BCG vaccination complicates the interpretation of TB skin tests. However, OPV and BCG vaccine recipients who are in the United States as visitors or immigrants might present their health care providers with complex medical issues related to vaccines other than those recommended by CDC and the American Academy of Pediatrics.

A boy from India aged 7 months was brought to a community hospital emergency department in San Antonio, Texas, in early July 2013. His parents reported that he had a 6-week history of intermittent fever associated with a draining skin lesion over the left deltoid at the site of BCG vaccination. The child was diagnosed with acute lymphadenitis, prescribed trimethoprim/sulfamethoxazole, and sent home.

The child again was brought to the emergency department with increased irritability and decreased movement of the left leg, and was admitted to the hospital for further evaluation. The child appeared tired and anxious but was responsive to touch. The physical examination was negative for meningeal signs, no evidence of respiratory distress was found. A firm, mobile, tender, 2×2-cm mass was palpated under the left axilla. The child did not move his left leg spontaneously or in response to pain. Deep tendon reflexes were absent in the left leg and diminished in the right. Decreased anal sphincter tone was noted. The rest of the physical examination was noted as normal for age.

Laboratory, imaging, and microbiologic studies were conducted (Table). Immunology evaluation revealed immunoglobulin (Ig) M and IgA levels to be extremely low, and IgG level low, reflecting waning maternal antibody. B and T cells were absent; however, NK cells were present. Magnetic resonance imaging revealed abnormal signals in the cervical and lower thoracic spinal cord and the cauda equine, suggesting the presence of an encephalitic or postinfectious demyelinating process. Viral cultures from stool specimens grew an enterovirus, which was confirmed by polymerase chain reaction.

With the history of two additional OPV vaccinations during national immunization days in India, a diagnosis of VAPP was considered. The stool culture was subsequently identified as iVDPV type 1 (iVDPV1). The nonrecombinant iVDPV1 isolates had 10- 12 nucleotide substitutions from Sabin 1 vaccine virus in the 906- nucleotide VP1 capsid protein coding region. This result is consistent with initiation of a period of prolonged virus replication after receipt of the first OPV dose, based on the rate of evolution of approximately 1% per year (6), although other potential sources of secondary exposure are possible. The sequences had one amino acid substitution in neutralizing antigenic site 1 and one amino acid substitution in neutralizing antigenic site 3a, compared with Sabin 1 virus. An axillary lymph node biopsy showed evidence of acute and chronic inflammation with the presence of macrophages. Blood culture identified *M. bovis*, confirming a diagnosis of BCG-osis (disseminated BCG infection). Genetic studies eventually confirmed the diagnosis of RAG-1 deficient SCIDS with homozygous mutation. Family history revealed the child’s older sibling died in infancy after rotavirus vaccination. Parental consanguinity and recurrent pregnancy losses in the mother were also reported. Chromosome microarray yielded homozygosity for >10% of the genome. The child progressed to respiratory distress during further observation. After consultation with multiple specialist physicians and with ethics committee review, the family chose to withdraw support, and the child died shortly thereafter.

## Discussion

Live, attenuated vaccines have had substantial impact in reducing or eliminating endemic infectious diseases but their administration is not without some risk. Live viral vaccines are contraindicated in persons with immune deficiencies, and this is part of the rationale for newborn screening for SCIDS. VDPVs can emerge to cause polio outbreaks in areas with low OPV coverage and can replicate for years in persons who are immunodeficient (7).

When these risks outweigh those of endemic disease, replacement of OPV by IPV is appropriate, as occurred in the United States after 1999. As the incidence of polio declines worldwide, similar considerations might apply in other countries. In one prospective study of 942 children and adults from Sri Lanka with symptoms suggestive of underlying immune disease, five patients were identified as having stool shedding of all three types of vaccine-strain poliovirus (8). Three of the five patients had been identified as having SCIDS, and none survived the first year of life. In a study involving patients from Tunisia with primary immunodeficiencies, polioviruses were detected in six patients, and all isolates were vaccine-related (9).

Use of OPV in India and Nigeria has led to decline in poliovirus transmission, which contributes to interruption of wild poliovirus globally, but the risk associated with OPV is VAPP. According to the National Polio Surveillance Project, a collaboration between India and the World Health Organization, five cases of VDPV infection were reported in India in 2013 (10). All cases were attributed to immunodeficiencies, but the case described in this report is the first in which SCIDS was confirmed by molecular genetic analysis. Similarly, although the use of BCG in countries with high prevalence of TB helps to prevent tuberculous meningitis and miliary disease, and is a highly cost-effective intervention against severe childhood TB infection, its use is not recommended in the United States because of low risk for infection with TB and the fact that BCG vaccination complicates the interpretation of TB skin tests.*

International travel carries a small risk for importation of live, attenuated vaccine organisms into the United States with attendant clinical consequences. Because vaccine schedules vary based on different public health considerations in different parts of the world, it is imperative that U.S. pediatricians be thorough and careful to know the immunization and family history of foreign-born children. In doing so, vaccine-related diseases, such as polio, can be considered in the differential diagnosis, and appropriate diagnostic specimens can be collected. By being vigilant, vaccine-associated diseases can be diagnosed early, the spread of disease can be prevented by immunization of exposed persons in the community and among contacts, and appropriate treatment can be given in a timely manner to minimize suffering and reduce morbidity and mortality.

What is already known on this topic?

Routine use of oral poliovirus vaccine was discontinued in the United States in the late 1990s, when the number of vaccine-associated paralytic polio cases exceeded the number of endemic cases. Endemic polio has not been eliminated worldwide. Thus, some countries continue to administer oral poliovirus vaccine.

What is added by this report?

In 2013, an immigrant to the United States, aged 7 months, was diagnosed with severe combined immune deficiency, paralytic poliomyelitis caused by a Sabine vaccine strain type 1 virus, and disseminated bacille Calmette-Guérin (BCG) infection (BCG-osis). The infant had been vaccinated with oral poliovirus vaccine and BCG in India.

What are the implications for public health practice?

Primary health care providers in the United States should recognize the potential for live viral vaccine diseases, such as vaccine-associated paralytic poliomyelitis and BCG-osis, in foreign-born children recently arrived from abroad. By being vigilant, vaccine-associated diseases can be diagnosed early, the spread of disease can be prevented by immunization of exposed persons in the community and among contacts, and appropriate treatment can be given in a timely manner to minimize suffering and reduce morbidity and mortality.

## Figures and Tables

**TABLE:** Laboratory, imaging, and microbiologic study results for a male patient aged 7 months recently immigrated from India who was brought to a hospital emergency department — San Antonio, Texas, July 2013

Type of study	Results
**Laboratory**	
CBC	ALC-216 cells/mm^3^
Lymphocyte subsets	CD3 = 6 cells/mm^3^; CD4 = 2; CD8 = 0; CD19 = 1; CD16/56 = 189
HIV 1/2	Negative
Immunoglobulins	IgA undetectable; IgM undetectable; IgG 140 mg/dL
CSF	83 WBCs/mm^3^; 50% PMNs; 42% MNCs; 2% lymphocytes; protein = 48 mg/dL; glucose = 49 mg/dL
**Imaging**	
Chest radiograph	Normal
Brain and spine MRI	8-mm lesion in right cerebral peduncle; prominent abnormal T2 weighted signal at cord T11 level on the left; additional abnormal signal and contrast enhancement of several nerve roots
Chest, abdomen, pelvis CT	Enlarged lymph nodes: left supraclavicular, left axilla, retroperitoneal
**Microbiology**	
Blood	No bacterial growth at 48 hrs; later positive for AFB identified as *Mycobacterium bovis*/BCG
CSF	Negative bacterial meningitis screen and Gram stain; negative fungal smear and culture; negative PCR for HSV-1, HSV-2, and CMV
Stool	Enterovirus isolated; identified as iVDPV1
Lymph node FNA	AFB stain positive; identified as *M. bovis*/BCG

**Abbreviations:** CBC = complete blood count; ALC = absolute lymphocyte count; HIV = human immunodeficiency virus; IgA = immunoglobulin A; IgM = immunoglobulin M; CSF = cerebrospinal fluid; WBCs = white blood cells; PMNs = polymorphonuclear neutrophils; MNCs = mononuclear cells; MRI = magnetic resonance imaging; CT = computed tomography; AFB = acid-fast bacilli; BCG = bacille Calmette-Guérin; PCR = polymerase chain reaction; HSV = herpes simplex virus; CMV = cytomegalovirus; iVDPV1 = immunodeficiency-associated vaccine-derived poliovirus type 1; FNA = fine-needle aspiration.

## Footnotes

* Additional information available at http://www.cdc.gov/tb.
